# Roderigo Lopez, Physician-in-Chief to Queen Elizabeth I of England

**DOI:** 10.5041/RMMJ.10306

**Published:** 2017-07-31

**Authors:** George M. Weisz, Donatella Lippi

**Affiliations:** 1 School of Humanities (Program in History of Medicine), University of New South Wales, Sydney, Australia; 2 School of Humanities, University of New England, Armidale, New South Wales, Australia; 3 Center for Medical Humanities, Department of Experimental and Clinical Medicine, University of Florence, Italy

**Keywords:** Dr Roderigo Lopez, ethics, history of medicine, Jewish history, persecution

## Abstract

Roderigo Lopez, former Physician-in-Chief to Queen Elizabeth I of England, was a controversial figure in his time and continues to be the subject of controversy. Much has been written about his religious practice, politics, and guilt, or lack thereof, with regard to charges of treason to the Crown. However, the fact remains that Lopez was the only physician to the Crown to be sentenced to death. All evidence points to an anti-Semitic mindset that played in the background. Yet Lopez so endeared himself to the Queen that although he was indeed sentenced to death, almost all of his property was restored to his family. This brief paper pays tribute to the Jewish physician, Roderigo Lopez, whose story was indeed a triumph over prejudice, despite his fate.

## INTRODUCTION

Worldwide, the history of medicine, like all of history, includes the history of people. Advances in medicine, in general, were accompanied by an enhanced status of the doctors facilitating that advancement—as well as that of the ethnic group to which they belonged. The opposite, however, seems to have been true of successful Jewish doctors. Their efforts rarely advanced the status of their own people up until the Enlightenment. Even after their emancipation in the nineteenth century, successful Jewish doctors have had little impact on how the world relates to Jews.

Jewish populations have been exposed to persistent and intense persecution throughout history. The Jewish people have endured restrictions in ownership, mobility, agriculture, and industry, leading to increased pressure to accept opportunities edging on dubious legality, with devastating results. One exception has been in the study of medicine and the practice of the art of healing, with strong evidence throughout history that Jewish physicians have been held in high regard.^1^

This is evidenced by the hypocritical attitude of monarchs, ecclesiastical rulers, and political leaders, who—whilst proclaiming anti-Jewish rules, preaching anti-Jewish sermons, introducing the Inquisition, establishing ghettos in Germany and Russia, and ordering expulsions from England, Spain, France, and Portugal—continued to retain the services of their trusted Jewish physicians.^1^ For example, Friar Roger Bacon (1220–1292), English philosopher and theologian, stated:

Christian physicians were ignorant in comparison with their Jewish colleagues, because they lacked knowledge of the Hebrew and Arabic in which most of the medical works were written.^1^

It was during Friar Bacon’s era, in 1290, that King Edward I of England expelled the Jews; this exile would last 366 years.^2^ The libertarian rights of the Magna Carta were not applied to the Jews. The case of the Portuguese physician, Roderigo Lopez, a refugee to England, was therefore an example of “triumph over prejudice” and is the topic of this paper.^3^–^5^

## BACKGROUND INFORMATION

Roderigo Lopez was born in 1524 (or 1525) in Crato, Portugal into a converted, or crypto-Jewish, family. He studied Medicine in Coimbra, graduating in 1544, when *auto-da-fe* (burning at the stake in public) was practiced. Suspected of secretly practicing Judaism, Lopez was driven out of Portugal by the Inquisition.^6^,^7^ He subsequently ended up in London and changed his name to Ruy Lopez (or Lopes).^4^,^5^

The rules of admitting physicians to medical practice in England were such that all, except graduates of Oxford or Cambridge universities, had to pass an assessment by an examining committee. No record confirming this assessment of Lopez has been found; however, it is assumed that it must have been awarded, since in 1567 Dr Lopez was admitted as the first regular physician to practice at St Bartholomew’s hospital and subsequently became a Fellow of the Royal College of Physicians. Soon his reputation assured his success amongst the highest classes of society. There is much discussion as to Lopez’s practice of religion: some insist he was a practicing Christian,^8^,^9^ although others assert he observed Judaism in secret;^10^ clearly, his Jewish ethnicity was never in question.^11^,^12^

## MEDICAL PRACTICE OF RODERIGO LOPEZ

Lopez practiced Galenic medicine and was skilled in diet, purges, and phlebotomies. A contemporary of Lopez, Gabriel Harvey, wrote that Lopez was one of the most learned and expert physicians at the Court, but attributed his success to “Jewish practice.”^13^,^14^ However, another equally famed contemporary, William Clowes, was deeply impressed by Lopez’s skills, particularly as a surgeon, and his recommendations for diets, purges, and bleeding.^5^ Lopez also had an affinity toward prescribing medicinal concoctions, such as “arceus apozema,” which may well have included anise and sumac berries, although the exact recipe is no longer known.^15^

Anise seeds are often prescribed as an aromatic tea and are recognized for their calming effect in respiratory ailments and their potent anti-colic effect in intestinal disorders as well as in dysmenorrhea.^16^,^17^ Today, anise is recognized to have a variety of beneficial effects and has been widely researched for its antioxidant, analgesic, and anticonvulsant properties, to name a few.^18^

Sumac was used for cooking and in a berry lemonade reputed to help with digestive ailments. Today it too is recognized for its antioxidant properties.^19^

The positive effects of anise and sumac may well have been recognized by Dr Lopez, as he prescribed their use many years before an official description of anise appeared in *The Herball or Generall Historie of Plantes*, printed in 1597.^20^ Three years after he entered her service, the Queen of England granted Lopez a monopoly for importing sumac and anise to England in 1589.^9^,^21^

## FROM ACCEPTANCE TO PERSECUTION

Dr Lopez enjoyed many years of success in England, and in 1584 he was named Physician-in-Chief to Queen Elizabeth I. However, despite his powerful friends, Lopez also gained powerful enemies, including the Earl of Essex, protector and intimate of the Queen. Perhaps it should not be surprising that Lopez would eventually find himself persecuted. As LeBlanc points out, Lopez achieved an extraordinarily high position at the English Court—a position no “Iberian, suspected Catholic, or suspected Jew should have found in Early Modern England …”^9^

The Earl of Essex was angered by Lopez’s unethical disclosure that the Earl had venereal disease, although it is unclear whether this disclosure was intentional or not. A dramatic sequence of events unfolded, and Dr Lopez was charged with conspiracy to poison the Queen, leading to his trial and conviction.^9^,^12^,^22^–^24^ The execution was delayed for some three months by the doubting monarch.^22^,^25^ Accused of being involved in a “political plot,” Lopez was charged with treason. He was executed in June 1594 in front of a large and jubilant crowd, to shouts of “hang the Jew.” At that time, victims of execution were hanged, drawn, and quartered.^26^ Nevertheless, even in death, Lopez proved victorious. Queen Elizabeth apparently doubted his guilt and exercised a rare option to restore most of Lopez’s property to his family.^10^

It is beyond the scope of this paper to discuss the political intrigue surrounding Lopez’s arrest, trial, and execution; these issues have been debated at great length elsewhere. However, there is strong evidence that anti-Semitism had a role in his fate. During the trial it was recorded that, “Lopez, *like a Jew* [emphasis added], did utterly with great oaths and execrations deny all …”^12^ This record makes the anti-Semitic bias against Lopez quite clear.

## DISCUSSION

The story of Roderigo Lopez is initially one of triumph over prejudice, despite the anti-Jewish demonstration at his execution.^11^,^12^ It has been hypothesized that Lopez was the figure who inspired the character of Shylock in Shakespeare’s “The Merchant of Venice,” which was written not long after the Lopez trial in 1596 and first performed in 1605.^27^

The authors believe it is important to recognize and acknowledge the important role Jewish physicians have played in history. To date, only a fraction of physicians’ lives and their contributions have been published relating to the medieval and early modern periods, namely the Renaissance, Baroque, and beyond.^28^,^29^

Their lives were not easy, yet their contributions were quite notable. Their successes were often a triumph over the prejudice driven by the fervent religious ideology in Europe of that time. The hypocrisy of those seeking the services of Jewish physicians is astonishing; on the one hand there was a deep and often public appreciation for their medical skills, but on the other hand they were the victims of strong prejudice based on their forefathers’ perceived sins. Yet their medical expertise is implied by the status of their clientele, as shown in Table 1.

**Table 1:** Partial List of Jewish Doctors with High-Profile Clients (13–18^th^ Centuries).

Century	Name	Client	Notes
12^th^	Alfakhar^1^	King Ferdinand, Toledo, Spain	
13^th^	Isaac Maestro Gaio^1^	Physician to Pope Boniface VIII Physician to Pope Nicholas IV	Pope Nicholas IV forced the Jewish population to wear yellow badges^1^
13^th^	Elias Sabot^1^,^25^	King Henry IV	King Henry IV defied King Edward’s Jewish exile order of 1290^2^
14^th^	Angelo de Manuelle^1^	Physician to Pope Boniface IX	
14^th^	Antonio Lopez^1^	King Joao (John) III, Portugal	King Joao III initiated the Portuguese Inquisition
15^th^	Isaac Cordoza^1^	King Philip IV, Madrid	
15^th^	Eliahu Montalto^29^	Queen Maria de Medici-Bourbon	
16^th^	Elias Ben Isaac Lattes^1^	Borgia Pope Alexander VI Medici Pope Leo X	
16^th^	Roderigo de Castro^1^	King of Denmark	
16^th^	Benedict de Castro^1^	Queen of Sweden	
Mid 16^th^	David de Pomis^1^	Pope Paul IV	Pope Paul IV was reputedly the fiercest anti-Jewish pontiff; he established the Roman Ghetto and issued anti-Jewish encyclicals^1^
17^th^	Jean Astruc^1^,^30^	King Louis XV, France	
17^th^	Jean Baptiste Silva^1^	Grand Duke of Bavaria Prince Henry de Conde, France Voltaire	Voltaire was well-known for ambivalent views on Jews^1^
17^th^	Benjamin Mussafia^1^	King Christian IV, Denmark	
18^th^	Antonio Ribeiro Sanchez^1^	Empress Ann of Russia Empress Elizabeth of Russia Empress Catherine II of Russia	All three Empresses imposed geographical restrictions on Jews in Russia

The triumph of Jewish physicians over prejudice must be attributed to their medical skill. There is no other explanation for their acceptance by those who, in some cases, were strong proponents of anti-Semitism.

In light of the tendency of humankind to rewrite history to support current, politically correct perspectives, it is important to maintain an accurate historical record of the contributions and efforts of Jewish physicians throughout history, and to take note of the persecution and injustice under which these doctors worked. While medical practice has markedly progressed over the centuries, the manner in which physicians and scientists themselves have been treated has not changed—in particular, but not only, Jewish physicians.^31^ Indeed in the last and this current century, physicians have been killed to ensure they would not provide medical care for the perceived enemy.^23^ Perhaps it is not surprising that the world remains silent to their plight, since throughout history the persecution and injustice of nations to Jewish people, including physicians, continue to be silenced. If we do not recognize the contributions of the Jewish people to the world—particularly with regard to medicine—who will be the next to go? As the German Martin Niemöller so aptly wrote:

First they came for the Socialists, and I did not speak out—
Because I was not a Socialist.
Then they came for the Trade Unionists, and I did not speak out—
Because I was not a Trade Unionist.
Then they came for the Jews, and I did not speak out—
Because I was not a Jew.

Then they came for me—and there was no one left to speak for me.
32
